# Assisted Vaginal Deliveries in Mothers Admitted as Public or Private Patients in Western Australia

**DOI:** 10.1371/journal.pone.0061699

**Published:** 2013-04-16

**Authors:** Kristjana Einarsdóttir, Fatima A. Haggar, Sarah Stock, Anthony S. Gunnell, Fiona J. Stanley

**Affiliations:** 1 Telethon Institute for Child Health Research, Centre for Child Health Research, University of Western Australia, Subiaco, Western Australia, Australia; 2 Centre for Health Services Research, School of Population Health, University of Western Australia, Crawley, Western Australia, Australia; 3 School of Women’s and Infant’s Health, University of Western Australia, King Edward Memorial Hospital, Perth, Western Australia, Australia; 4 Health and Wellness Institute, Edith Cowan University, Joondalup, Western Australia, Australia; Baylor College of Medicine, United States of America

## Abstract

**Background:**

Mothers delivering as private patients in Australia have a high rate of assisted deliveries, which could lead to adverse infant outcomes in this group of patients. We investigated whether the risk of adverse infant outcomes after assisted deliveries was different for mothers admitted as public or private patients for delivery, when compared with unassisted deliveries.

**Methods and findings:**

We included 158,241 vaginal, singleton, term birth admissions in our study where the infant was live born and without birth defects. The study population was identified from statutory birth and hospital data collections held by the Western Australian (WA) Department of Health. We estimated odds ratios and confidence intervals using logistic regression models adjusted for a range of maternal demographic, pregnancy and birth characteristics. Interaction was assessed by including interaction terms in the models. Outcomes included low Apgar scores at five minutes (<7), neonatal resuscitation and special care admission. Mothers delivering as private patients had an increased risk of assisted vaginal delivery compared with public patients (adjusted OR 1.74, 95% CI = 1.68–1.80). Compared with unassisted vaginal deliveries, assisted deliveries were associated with increased risk of Apgar scores at five minutes below 7 (OR 1.25, 1.08–1.45), neonatal resuscitation (OR = 1.69, 1.42–2.00) and admission to special care nursery (OR = 1.64, 1.53–1.76). The increased risk of neonatal resuscitation was higher for mothers admitted as private patients for delivery (OR = 2.13) than public patients (OR = 1.55, p^interaction^ = 0.03).

**Conclusions:**

Our results suggested that the high risk of neonatal resuscitation following assisted vaginal deliveries compared to unassisted is higher in private patients than public patients. Whether this phenomenon is due to the twofold higher rate of assisted vaginal deliveries in this group of patients or a higher rate of fetal indications for assisted vaginal delivery remains to be answered.

## Introduction

In Australia, women can receive antenatal care as public patients, with the care provided by rostered midwives, residents, registrars and staff obstetricians in public hospitals, or as privately funded patients where the antenatal care is led by private obstetricians with women delivering in either public or private hospitals. Public patients are covered by a national health insurance scheme known as Medicare, whereas private patients are treated at their own expense or at a subsidised cost through Private Health Insurance (PHI) [1], [2].

Rates of assisted vaginal delivery have been reported to vary greatly around the world [3] with higher rates (13%) seen in countries such as the United States and Australia [4], [5] and lower rates (5%) in some European countries [6]. Mothers holding private health insurance in Australia have been reported to experience a higher rate of instrumentally assisted deliveries and other obstetric interventions than other mothers [7], [8], [9]. For example, of all low risk deliveries in New South Wales, Australia, vacuum deliveries have been reported to be 7% in public patients and 11% in private patients [8]. Similar findings have been reported in the United States, where women with private insurance experience a higher rate of caesarean deliveries and episiotomies than women treated publicly [10], [11]. This may seem surprising given that mothers admitted as private patients for delivery are generally healthier than public patients, live in areas of lower socio-economic (SE) disadvantage, have fewer complications during pregnancy and are less likely to give birth to infants with adverse outcomes [8], [10], [11], [12], [13], [14], [15], [16], [17].

Assisted vaginal deliveries have been reported both nationally and internationally to be associated with increased risk of most adverse infant outcomes compared with unassisted vaginal deliveries [4], [18], [19]. Given the risk associated with this procedure and the great difference in rates between mothers who give birth as either public or private patients, it would be important to know whether the higher rate of assisted vaginal births in private obstetric patients could lead to worse infant outcomes in this group of mothers. Our aim was to answer this question by assessing the risk of neonatal complications following assisted vaginal deliveries compared with unassisted vaginal deliveries separately for mothers admitted as public patients and private patients for delivery in Western Australia (WA).

## Methods

### Ethics Statement

This study and the use of the de-identified data without patient consent was approved by the Human Research Ethics Committee of the WA Department of Health and performed in accordance with the Declaration of Helsinki.

### Data Information

In this study we used linked de-identified, administrative health data from the WA Midwives Notification System (MNS), the WA Hospital Morbidity Data Collection (HMDC) and the WA Birth Defects Registry provided by the Data Linkage Branch at the WA Department of Health. Included in the MNS data were all pregnancy and delivery details for all infants born alive or stillborn in WA during 1988–2008 at 20 weeks or greater gestation or with birth weight of at least 400 gm. The hospital data included all hospital admission information for each birth admission during 1988–2008 and the Birth Defects Registry included information on whether the infants had minor or major birth defects. For the study population included in this study, we restricted the data to vaginal, singleton, term births (37–41 completed weeks), where the infant was live born and without birth defects.

The MNS included information on the Index of Relative Socio-Economic (SE) Disadvantage (IRSD) and Accessibility/Remoteness Index of Australia (ARIA+). The IRSD values are based on information on household income, educational attainment and occupation from the Australian census conducted every five years and assigned to each collection district area in the state. The IRSD values were divided into quintiles for all analyses, with high scores reflecting low SE disadvantage in an area. The ARIA residential remoteness index is also calculated from census information every five years and reflects access to services in a collection district area. It was divided into major cities, inner regional Australia, outer regional Australia, remote Australia, and very remote Australia. The IRSD and ARIA values from the 1991, 1996, 2001, and 2006 censuses were assigned to each birth admission based on maternal area of residence at the time of birth.

The MNS also included information on pregnancy complications and labour and delivery complications. Pregnancy complications included pre-eclampsia, placental abruption, pre-labour rupture of membranes, and gestational diabetes. Labour and delivery complications included precipitate delivery, fetal distress, prolapsed cord, cord tight around neck, persistent occiput posterior, shoulder dystocia, failure to progress at >3 cm dilation, and previous caesarean section.

We categorised patient funding source to reflect two types of patients; those treated as public patients and those treated as private patients regardless of whether the birth occurred in a public or private hospital. Private patients were defined as those funded with PHI (98%) or who were self-funded (2%), as self-funded patients were classified as private patients at admission and similar proportion of both groups lived in low SE disadvantaged areas (83% and 87%, respectively). Public patients included those insured under the Australian Health Care Agreements or Reciprocal Health Care Agreements.

### Statistical Analysis

Differences in characteristics according to patient status were assessed using chi square tests of independence. We used a logistic regression model to estimate odds ratios and 95% confidence intervals for the risk of assisted vaginal delivery for mothers delivering as private patients compared with mothers delivering as public patients. Assisted vaginal deliveries included deliveries requiring the use of vacuum and/or forceps. The model was adjusted for year of birth, maternal age, parity, smoking during pregnancy, marital status, ethnicity (Caucasian/Indigenous/other), pre-existing medical conditions (asthma/hypertension/diabestes), socio-economic status (SES) quintiles, residential remoteness, gestation, pregnancy complications, analgesia during labour (none/gas or intramuscular/epidural/spinal), and whether the labour was induced. The predictive power of the model was R^2^ = 0.31. We assessed multicollinearity using the PROC REG procedure in SAS and found no evidence for collinearity between the explanatory variables.

We also used logistic regression models to assess the association between mode of delivery (vaginal unassisted/assisted) and the risk of adverse infant outcomes. Infant outcomes included Apgar score at 5 minutes (<7), neonatal resuscitation (endotracheal intubation or external cardiac massage) or admission to a special care nursery. The models were adjusted for year of birth, maternal age, parity, patient status (not in stratified analysis), hospital type (private/public/tertiary), smoking during pregnancy, marital status, ethnicity (Caucasian/Indigenous/other), pre-existing medical conditions (asthma/hypertension/diabestes), SES quintiles, residential remoteness, gestation, infant weight, pregnancy complications, labour and delivery complications, analgesia during labour (none/gas or intramuscular/epidural/spinal), and whether the labour was induced. The predictive power of the models was R^2^ = 0.09, R^2^ = 0.15, and R^2^ = 0.09, respectively. No evidence for collinearity was found between the explanatory variables included in the models. All analyses were first performed on the whole study sample and then stratified by patient status. This was done as the objective was to display the individual risks separately for private and public patients as opposed to directly comparing the two groups. Using the adjusted models for whole study population, we calculated p-values for interaction between mode of delivery (unassisted/assisted) and patient status (public/private) for the risk of adverse infant outcomes by adding an interaction term in the models (i.e. patient_status*mode_of_delivery). All analyses were performed using the statistical software SAS version 9.3 (SAS Institute Inc., Cary, NC, USA).

## Results

Of the 158,241 vaginal births included in this study, unassisted deliveries comprised 81% (128,245) and assisted deliveries 19% (29,996). Table 1 shows the characteristics of the study population for mothers delivering either as public (71%) or private patients (29%). Older mothers, mothers who did not smoke, mothers living in less disadvantaged areas, mothers who were less likely to give birth in week 40–41, mothers who gave birth to slightly larger infants and mothers who were induced or received epidural/spinal analgesia during labour were more likely to be admitted as private patients for delivery (Table 2). All differences were statistically significant (p<0.0001).

**Table 1:** Characteristics of 158,241^a^ WA vaginal births for mothers delivering as either public or private patients.

	Public (n = 112,222)	Private (n = 46,019)	Degrees of freedom	
	Mean (SD)	Mean (SD)		p-value^b^
Infant weight at birth (g)	3450.4 (462.6)	3474.6 (426.2)	92371	<0.0001
Maternal age (years)	27.4 (5.7)	31.2 (4.4)	109975	<0.0001
	n (%)	n (%)		
Maternal age (years)				
12–17	3,182 (2.8)	79 (0.2)		
18–30	75,009 (66.8)	19,676 (42.8)		
31–40	32,862 (29.3)	25,524 (55.5)		
41–50	1,169 (1.0)	740 (1.6)	3	<0.0001
Smoking during pregnancy				
No	82,931 (73.9)	43,531 (94.6)		
Yes	29,291 (26.1)	2,488 (5.4)	1	<0.0001
Area-based SES quintiles				
1 least disadvantaged area	14,712 (13.1)	17,273 (37.5)		
2	20,999 (18.7)	12,516 (27.2)		
3	22,889 (20.4)	8,168 (17.8)		
4	26,025 (23.2)	5,518 (12.0)		
5 most disadvantaged area	27,597 (24.6)	2,544 (5.5)	4	<0.0001
Gestation (completed weeks)				
37	6,377 (5.7)	2,663 (5.8)		
38	17,122 (15.3)	9,666 (21.0)		
39	24,299 (21.7)	12,107 (26.3)		
40	47,732 (42.5)	17,458 (37.9)		
41	16,692 (14.9)	4,125 (9.0)	4	<0.0001
Onset of labour				
Spontaneous	78,307 (69.8)	24,786 (53.9)		
Induction	33,915 (30.2)	21,233 (46.1)	1	<0.0001
Epidural during labour				
No	81,866 (73.0)	19,888 (43.2)		
Yes	30,356 (27.1)	26,131 (56.8)	1	<0.0001

a Restricted to vaginal, singleton, term births (37–41 completed weeks), where the infant was live born and without birth defects.

b Chi square test of independence.

**Table 2:** Risk of assisted vaginal delivery and fetal distress for mothers delivering as private patients compared with mothers delivering as public patients in a study population of 158,241^a^ WA vaginal births.

	Public patient (referent)	Private patient	Unadjusted model	Adjusted model b
	n (%)	n (%)	OR (95% CI)	OR (95% CI)
Assisted vaginal delivery	16,182 (14.4)	13,814 (30.0)	2.55 (2.48–2.61)	1.74 (1.68–1.80)
Fetal distress	13663 (12.2)	5235 (11.4)	0.93 (0.90–0.96)	0.78 (0.75–0.82)

a Restricted to vaginal, singleton, term births (37–41 completed weeks), where the infant was live born and without birth defects.

b Adjusted for year of birth, maternal age, parity, smoking during pregnancy, marital status, ethnicity (Caucasian/Indigenous/other), pre-existing medical conditions (asthma/hypertension/diabestes), SES quintiles, residential remoteness, gestation, pregnancy complications, analgesia during labour (none/gas or intramuscular/epidural/spinal), and whether the labour was induced.

The risk of assisted vaginal delivery for private compared with public patients is presented in Table 2. Assisted vaginal deliveries were over twice as likely in mothers admitted as private patients for delivery (30%), compared with public patients (14.4%), resulting in a 2.55 increased risk of assisted vaginal deliveries for private patients. After adjusting for maternal age, smoking status, SES, pre-existing medical conditions, pregnancy complications, epidural use and other factors, private patient status continued to be associated with the risk of assisted vaginal delivery, compared with public (1.74, 95% CI 1.68–1.80).

In Table 2 we also show the risk of fetal distress in private compared with public patients as fetal distress can in some cases be an indication for assisted vaginal delivery. Despite having an increased risk of assisted vaginal delivery compared with public patients, private patients were less likely to experience fetal distress during delivery, both before (0.93, 95% CI 0.90–0.96) and after covariate adjustment (0.78, 95% CI 0.75–0.82).

**Figure 1:**
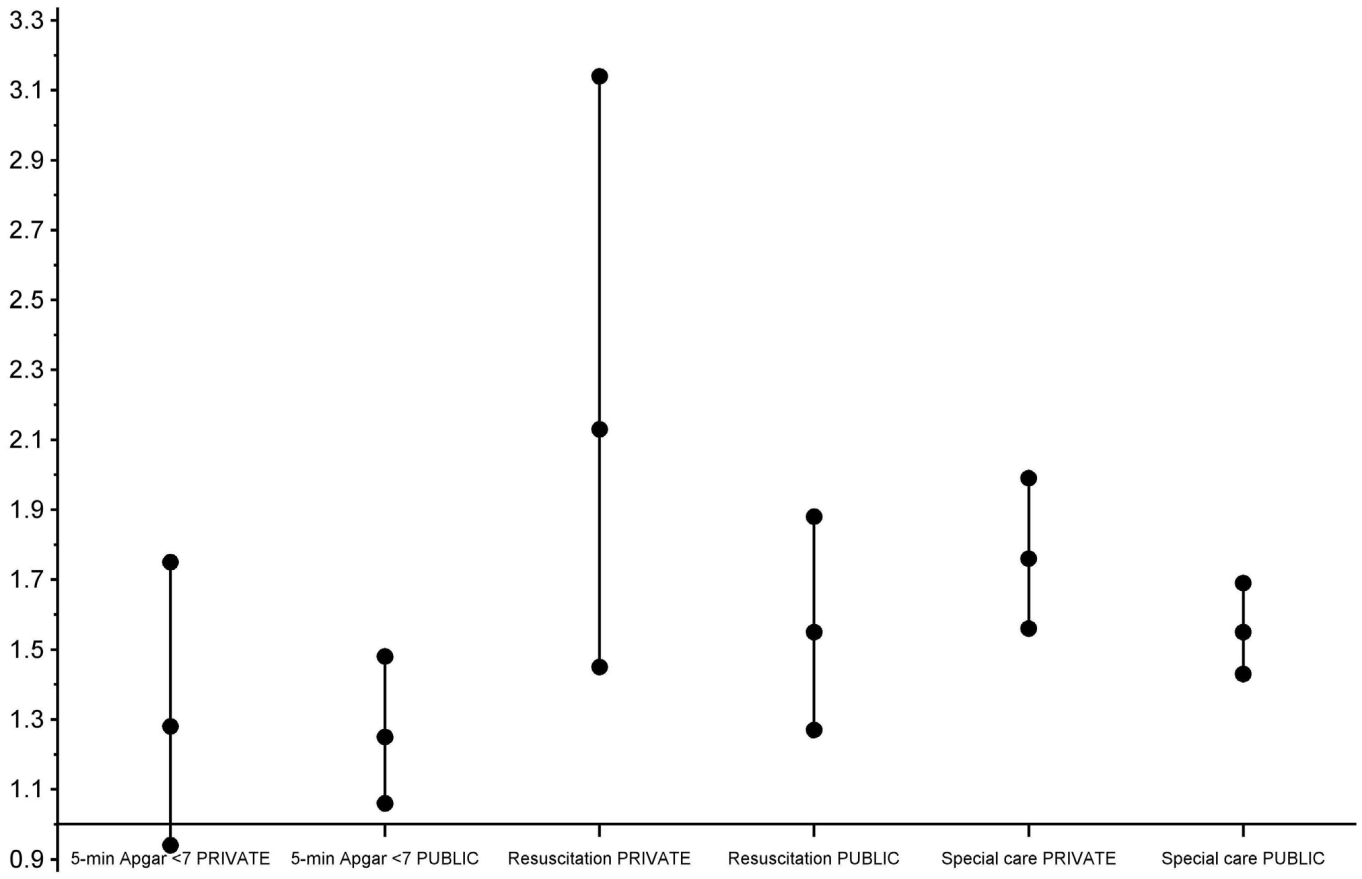
Risk of adverse infant outcomes for assisted vaginal deliveries compared with unassisted deliveries in 158,241 WA public and private patient birth admissions. X-axis represents odds ratios (middle black dots) and 95% confidence intervals (top and bottom black dots). Logistic regression models -adjusted for the same factors as in Table 3- were run separately for private and public patients to calculate separate odds ratios (ORs) for the risk of adverse infant outcomes for assisted vaginal deliveries compared with unassisted deliveries.

We show the association between mode of delivery and the risk of adverse infant outcomes after birth in Table 3. Compared with unassisted vaginal deliveries, infants born with assistance had an increased risk of all adverse infant outcomes, even after adjustment (ORs 1.25, 1.69 and 1.64). After stratification by patient status (Figure 1), we found no statistically significant difference between mothers admitted as public or private patients for the risk of low Apgar scores (p^interaction^ = 0.88) or special care admission (p^interaction^ = 0.22) following assisted deliveries compared with unassisted deliveries. However, the increased risk of neonatal resuscitation for assisted deliveries compared with unassisted was significantly higher in mothers admitted as private patients (OR = 2.13) for delivery than in mothers admitted as public patients (OR = 1.55) (p^interaction^ = 0.03).

**Table 3:** Risk of adverse maternal and infant outcomes after assisted vaginal delivery compared with unassisted vaginal for 158,241^a^ WA vaginal births.

	Unassisted Vaginal(referent)	Assisted vaginal	Unadjusted model	Adjusted model b
	n (%)	n (%)	OR (95% CI)	OR (95% CI)
Apgar score at 5 min <7^c^	863 (0.7)	404 (1.4)	2.02 (1.79–2.27)	1.25 (1.08–1.45)
Neonatal resuscitation^d^	476 (0.4)	347 (1.2)	3.14 (2.73–3.61)	1.69 (1.42–2.00)
Admission to special care	4,286 (3.3)	1,971 (6.6)	2.03 (1.93–2.15)	1.64 (1.53–1.76)

a Restricted to vaginal, singleton, term births (37–41 completed weeks), where the infant was live born and without birth defects.

b Adjusted for year of birth, maternal age, parity, patient status (not in stratified analysis), hospital type (private/public/tertiary), smoking during pregnancy, marital status, ethnicity (Caucasian/Indigenous/other), pre-existing medical conditions (asthma/hypertension/diabestes), SES quintiles, residential remoteness, gestation, infant weight, pregnancy complications, labour and delivery complications, analgesia during labour (none/gas or intramuscular/epidural/spinal), and whether the labour was induced.

c Apgar score at 5 minutes = 0–6.

d Endotracheal intubation or external cardiac massage.

## Discussion

This study investigated the rate of and outcomes after assisted vaginal deliveries in mothers admitted as public and private patients for delivery in WA. We found that private patients had twice the risk of assisted deliveries than public patients despite being less likely to experience fetal distress during delivery. Also, the high likelihood of neonatal resuscitation following assisted vaginal deliveries compared with unassisted, was significantly higher in private patients than public patients.

The rates of assisted vaginal deliveries for public and private patients delivering in WA reported in this study conformed to those previously published for NSW and Queensland [7], [8], although the rates for Queensland were considerably higher for public patients. This difference could have been due to the fact that their study did not include whole-population data, but was a sample of 242 pregnant women, whereas both our study and the NSW study included data from an entire state. Our results were also in line with previous findings which have suggested an increased risk of Apgar scores at five minutes below 4 [18], mechanical resuscitation [4] and admission to neonatal intensive care [19] following assisted deliveries compared with unassisted.

Our results also indicated that private patients have twice the risk of needing assistance during vaginal delivery than public patients. Previous findings have suggested that privately insured women in Australia have more likelihood of receiving episiotomy [9], a higher probability of caesarean or instrumentally assisted delivery [7], and a higher risk of forceps or vacuum delivery and of other obstetric interventions such as epidural anaesthesia, induction or augmentation [8], compared to women without private insurance. Similar results are reported in the international literature, where midwife-led care is associated with fewer obstetric interventions than other models of care [20]. It is likely that there are many reasons for this difference in intervention rates between mothers delivering as public patients and those delivering as private patients, including fear of litigation [21] and maternal request [22]. However, given that mothers delivering as private patients are generally healthier than public patients and have fewer complications during pregnancy [8], [10], [11], [12], [13], [14], [15], [16], [17], it seems unlikely that the increased intervention rate in private patients is due to maternal or foetal risk.

Given the high obstetric intervention rate in the private sector, it appears possible that our results are at least partly due to the higher rate of assisted deliveries in private patients. This is because even though neonatal resuscitation is more common in public than private patients for both unassisted and assisted deliveries, the higher rate of assisted deliveries and lower rate of unassisted deliveries in private patients results in a higher risk of neonatal resuscitation for these patients when assisted deliveries are compared with unassisted. Furthermore, for all infants who required resuscitations, 42% were born with assisted vaginal delivery. However for infants of private patients who required resuscitation, 66% were born with assisted delivery, but for public patients, only 35% were born with assisted delivery. This could mean that the mode of delivery did play more of a role in the indication for resuscitation for infants of private patients than for the infants of public patients.

However, the lack of information on indication for assisted vaginal delivery or neonatal resuscitation makes it difficult to establish the true reason for the observed association in our study. For example, we do not know the reason for the high assisted vaginal delivery rate in the private sector. It is possible that some indications such as fetal bradycardia and maternal fever are increased in this group of patients, which may lead to an increased need of neonatal resuscitation following birth. Despite not having information on indication for assisted vaginal delivery, we were able to investigate the frequency of fetal distress in public and private patients. Fetal distress is suspected when there is decreased fetal movement or increased or decreased fetal heart rate, particularly following contractions. Although this is a general indicator of the fetal condition, it gives some idea of the differences in assisted vaginal delivery indications between private and public patients. Our results indicated that despite being more likely to undergo assisted vaginal delivery than public patients, private patients were less likely to experience fetal distress during delivery. These findings should not be interpreted as all indications will be less likely in private patients, but they nevertheless suggest that the high assisted vaginal delivery rate in private patients is not entirely due to higher rate of indications in this group of patients.

Other possible reasons for the observed differences in neonatal resuscitation between private and public patients could be explained by differences in availability of medical staff at private and public hospitals. Neonatal intubation is commonly used by skilled senior paediatricians to assist in the clearance of airways with meconium and private hospitals are more likely to have paediatricians on hand to perform this task during care of the newborn instead of public hospitals since the latter are more likely to be staffed with resident medical officers or other junior staff.

### Strengths and Limitations

As randomised controlled trials are not feasible for the investigation of assisted vaginal deliveries, we used routinely and prospectively collected, population based observational data obtained from the statutory data collections of the WA Department of Health to investigate the study aims. The strength of this study is reflected in this use of large population based birth and hospital data as our data contained a near complete (99.98%) record of these events in the state. There are nevertheless some limitations related to using administrative health data, such as the lack of information on important variables that could confound the observed associations. In this case, we did not have information on weight gain during pregnancy, body mass index at the beginning of pregnancy, indication for assisted vaginal delivery, station of application of forceps or vacuum, years of clinical experience of care providers or other similar factors. However, we believe we were able to minimize residual confounding in this study through both direct and indirect adjustment by adjusting our analysis for all relevant maternal demographics, and pregnancy and labour characteristics, including pregnancy complications, SE disadvantage, labour and delivery complications, and analgesia.

The study cohort we used in this study included both primiparous and multiparous mothers as well as all breech deliveries. In order to assess whether including both primiparous and multiparous mothers was the correct decision, we performed sensitivity analyses restricting the study population to only primiparous mothers. The results for primiparous mothers were almost identical to the results for both primiparous and multiparous mothers, albeit with wider confidence intervals, and we thus felt assured that the validity of our results was not compromised. Furthermore, we felt confident in our decision to include all breech deliveries despite that the proportion of breech deliveries was different between public (0.23%) and private (0.13%) patients. This was reinforced by the fact that our results were almost identical after exclusion of all breech deliveries from the data.

### Conclusions

We studied the rate of assisted deliveries in mothers delivering as private patients and public patients in WA and whether the differences in rates affected the risk of adverse infant outcomes following assisted deliveries compared with unassisted vaginal deliveries in private versus public patients. Our results suggested that the high risk of neonatal resuscitation for assisted vaginal delivery compared with unassisted vaginal deliveries is higher in private patients than public patients. Whether this phenomenon is due to the twofold higher rate of assisted vaginal deliveries in this group of patients or a higher rate of fetal indications for assisted vaginal delivery remains to be answered.
